# Family and school support systems associated with mental health in Chinese migrant and non-migrant children

**DOI:** 10.3389/fpsyg.2026.1880778

**Published:** 2026-08-19

**Authors:** Meihua Guo, Yinyin Zhao, Jinhua Zhou, Liangyi Li, Chengchieh Li

**Affiliations:** 1 Fujian Province Key Laboratory of Applied Cognition and Personality, Institute of Applied Psychology, School of Education and Psychology, Minnan Normal University, Zhangzhou, China; 2 Tianjin Lei Feng Primary School, Tianjin, China; 3 Department of Psychology, Asia University, Taichung, Taiwan

**Keywords:** mental health, migrant children, parent–child communication, peer relationships, perceived teacher support

## Abstract

**Background:**

Previous research has suggested that migrant children often have weaker family and school support and poorer mental health than non-migrant peers, although recent evidence indicates that these disparities may be narrowing. Rather than focusing solely on mean-level group differences, the present study examined whether the pathways linking family and school support with mental health differ between migrant and non-migrant children.

**Methods:**

Guided by ecological systems theory, this study investigated the associations among parent–child communication, peer relationships, perceived teacher support, and mental health in 600 Chinese primary school students (303 migrant and 297 non-migrant children). Group differences in descriptive statistics, correlations, and regression coefficients were examined, followed by multi-group mediation analyses.

**Results:**

Migrant and non-migrant children did not differ significantly in mean levels of parent–child communication, peer relationships, perceived teacher support, or mental health. Parent–child communication was positively associated with mental health in both groups, whereas the association between peer relationships and mental health was stronger among migrant children than among non-migrant children. Mediation analyses further indicated that peer relationships served as the most robust indirect pathway linking parent–child communication to mental health, and this indirect pathway was significantly stronger among migrant children. By contrast, pathways involving perceived teacher support were comparatively weak and were not consistently supported across groups.

**Conclusion:**

These findings suggest that the pathways linking family-, peer-, and teacher-related factors with mental health differ across migration contexts. The results highlight the importance of considering both family- and peer-related factors when examining mental health in children with different migration experiences.

## Introduction

With rapid urbanization and large-scale internal migration, China has experienced a substantial increase in the population of migrant children, with more than 12 million enrolled in compulsory education in recent years (Ministry of Education of the People’s Republic of China, 2026). Within this sociocultural context, migration often involves changes in family structure, residential stability, and school environments, which may influence children’s access to social support across family and school settings.

Prior research has documented elevated risks for emotional and behavioral difficulties among migrant children, including insecurity, loneliness, and reduced well-being (Sun and Han, 2018; Tang et al., 2024; Zhang et al., 2020; Zhou et al., 2022). However, findings are not entirely consistent. Improvements in urban educational access and inclusion policies appear to have narrowed some developmental disparities in recent years (Han, 2023). These mixed results suggest that simple mean-level comparisons between migrant and non-migrant children may obscure more nuanced patterns in how different sources of social support are related to mental health.

Rather than focusing solely on whether migrant children exhibit poorer mental health on average, it may be more informative to examine how family- and school-based support systems are linked to mental health across migration contexts.

### An ecological systems perspective on children’s mental health

Ecological systems theory, proposed by Bronfenbrenner (1979), conceptualizes child development as occurring within nested and interacting environmental systems. Within this framework, children’s mental health is shaped by multiple ecological systems, including microsystems (e.g., family, peers, school), mesosystems (interrelations among microsystems), and the chronosystem, which captures the role of life transitions and sociohistorical change over time (Bronfenbrenner, 1979; Eriksson et al., 2018; Tudge et al., 2022). Migration experiences can be understood as a chronosystemic condition that may reorganize children’s ecological environments (Lou et al., 2025; Tudge et al., 2022), which may alter family communication patterns, peer integration opportunities, and perceptions of teacher support. Consequently, migration may shift the relative salience of family-based and school-based support systems in relation to children’s mental health.

From this perspective, examining how family, peer, and teacher supports are linked to mental health—and whether these association patterns differ by migration experiences—provides a more developmentally grounded understanding of children’s adjustment.

### Parent–child communication within the family microsystem

Among microsystems, the family represents the most enduring and proximal developmental context. Parent–child communication, defined as the ongoing exchange of information, emotions, and perspectives between parents and children, serves as a central mechanism linking the family environment to psychological adjustment (An, 2004; Barnes and Olson, 1985; Liu et al., 2022). High-quality parent–child communication is consistently associated with better emotional regulation and lower levels of internalizing problems (Li and Mo, 2024; Wagner et al., 2025; Yuan et al., 2024). However, in the migration context, the function of this communication may vary. While some studies report lower levels of communication in migrant families (Liu et al., 2022; Tang et al., 2024), others find family resilience (Han, 2023; Meehan et al., 2003; Zhang et al., 2009).

These mixed findings highlight the importance of moving beyond direct correlations and considering whether parent–child communication may exert an indirect association with children’s mental health through other developmental contexts, such as peer relationships and teacher support.

### Peer relationships and perceived teacher support within the school microsystem

Beyond the family, peer relationships constitute a cornerstone of children’s socialization (Parker and Asher, 1987; Zou, 1998). High-quality school-based peer relationships provide emotional support, a sense of belonging, and opportunities to practice social skills, thereby promoting social competence, identity development, and psychological resilience (Allen et al., 2022; Haddow et al., 2021; Wentzel and Caldwell, 1997). Evidence indicates that migrant children may experience lower levels of peer acceptance and reduced friendship quality within school contexts compared with their non-migrant peers, and they are more likely to encounter peer neglect or social exclusion in classroom and school-based interactions (Li and Jiang, 2018; Zhu et al., 2013).

Perceived teacher support represents another important aspect of the school microsystem. It refers to students’ perceptions that teachers provide academic guidance, emotional care, and respect, thereby fostering a supportive and structured learning environment (Babad, 1990; Ouyang, 2005). Research has shown that perceived teacher support is correlated with students’ academic motivation, school engagement, and mental health (Tao et al., 2022), with some evidence suggesting that teacher support is particularly relevant for the academic experiences of migrant children (Liu, 2024).

Although prior research has documented the independent correlations of peer relationships and teacher support with mental health, these factors are often examined in isolation. From an ecological perspective, experiences within the school microsystem are often interconnected and embedded in broader family-school linkages. Positive parent–child communication may foster emotional security, trust, and social competence, which can support children’s ability to establish more adaptive peer relationships (Li and Mo, 2024; Liu et al., 2022; Sroufe, 2005). Peer experiences may then shape children’s day-to-day classroom adjustment, including their sense of belonging, emotional security, behavioral engagement, and willingness to participate in classroom interactions. These school experiences are likely to influence not only children’s behavior toward teachers but also how they perceive and interpret teachers’ responsiveness and support. From this perspective, peer relationships may mediate the association between family processes and children’s perceived teacher support at school.

### Migration as a chronosystem-related life transition

Ecological systems theory also highlights the chronosystem, which refers to the patterning of environmental events, life transitions, and broader sociohistorical change over time (Bronfenbrenner, 1979). From this perspective, migration can be understood as a chronosystem-related life transition because it may alter children’s residential, family, and school environments and, in turn, reshape the developmental contexts in which adjustment unfolds.

Importantly, migration may influence not only children’s levels of support, but also the relative salience of support from different ecological contexts. In relatively stable family and residential contexts, family processes may remain the most consistent source of emotional support and therefore a central correlate of children’s mental health. By contrast, under migration-related transitions, disruptions in family routines, parental availability, residential continuity, or school belonging may increase children’s reliance on school-based relationships as accessible sources of belonging and day-to-day emotional support (Li and Jiang, 2018; Liu, 2024; Meehan et al., 2003; Zhang et al., 2009; Zhu et al., 2013). At the same time, migration may also alter how children perceive and engage with teacher support within the school context.

Accordingly, migration should not be conceptualized only as a background characteristic associated with mean-level differences in support or mental health. It may also condition the strength and configuration of associations among parent–child communication, peer relationships, perceived teacher support, and mental health. Examining these pathways across migrant and non-migrant children may therefore provide a more developmentally grounded understanding of how family and school support systems operate under different ecological conditions.

### The present study

Grounded in ecological systems theory, the present study examined how family- and school-based support processes are associated with children’s mental health and whether these associative patterns differ between migrant and non-migrant children. Specifically, parent–child communication was conceptualized as a family-microsystem process, whereas peer relationships and perceived teacher support were conceptualized as interrelated school-microsystem processes. Migration was treated as a chronosystem-related life transition that may condition the relative salience and interconnections of these support pathways.

Accordingly, we tested a serial mediation model in which peer relationships and perceived teacher support sequentially mediated the association between parent–child communication and mental health, while also allowing for a direct pathway from parent–child communication to perceived teacher support. We further used a multi-group analytic strategy to examine whether the strength of these pathways differed between migrant and non-migrant children. This approach was intended to move beyond simple mean-level comparisons and to clarify whether migration is associated with different configurations of family and school support in relation to children’s mental health. Within this framework, the following hypotheses were proposed:

*H1*: Parent–child communication, peer relationships, and perceived teacher support are significantly associated with children’s mental health, and the strength of these associations differs between migrant and non-migrant children.

*H2*: Peer relationships and perceived teacher support mediate the association between parent–child communication and children’s mental health.

*H3*: The mediation pathways linking parent–child communication and mental health differ between migrant and non-migrant children.

## Method

### Participants and procedure

We recruited participants from three public primary schools in northern China. These schools were selected based on two criteria: (1) a relatively high proportion of migrant children (approximately 40–55% of the student body, according to school administrative records), and (2) accessibility and willingness to participate in the study. The city itself is characterized by a substantial influx of migrant families from surrounding rural areas, making it a suitable setting for examining migration-related questions in an educational context. Given practical constraints of school access and the natural classroom structure, a cluster-based convenience sampling strategy was adopted, with intact classes serving as the primary sampling units. Students in Grades 3 to 6 were invited to participate because children at this stage have developed sufficient reading comprehension to complete the questionnaires independently. They are also transitioning from lower to upper primary school, a developmental period during which peer and teacher relationships become increasingly salient.

A total of 650 paper-and-pencil questionnaires were distributed and administered in classroom settings. The study protocol was approved by the institutional ethics committee (Psychology and Education Research Ethics Committee, Minnan Normal University; Approval No. 2026-03-02). Written informed consent was obtained from school administrators, teachers, and students’ guardians prior to data collection. Students were informed of the purpose of the study, assured of anonymity, and participation was voluntary. Questionnaires were completed anonymously during class time under the supervision of trained research assistants.

Six hundred questionnaires were valid and included in the final analyses (valid response rate = 92.3%). Invalid responses included those with more than 10% missing data or selecting the same option for almost all items. The final sample consisted of 303 migrant children and 297 non-migrant children. Among the migrant children, 164 were boys (54.1%), and 139 were girls (45.9%). Among the non-migrant children, 157 were boys (52.9%), and 140 were girls (47.1%).

### Measures

#### Parent–child communication

Parent–child communication was assessed using the Parent-Adolescent Communication Scale (Barnes and Olson, 1982/1985), which was translated and adapted for Chinese children by An (2004). The scale consists of 20 items and comprises two subscales: open communication and communication problems. All items were rated on a 4-point Likert scale. Reverse-coded items were reverse scored prior to analysis, and composite scores were calculated, with higher scores indicating better quality of parent–child communication. A confirmatory factor analysis indicated a good model fit, *χ*^2^(169) = 335.18, *p* < 0.001, *RMSEA* = 0.040 (90% CI [0.034, 0.047]), *CFI* = 0.939, *TLI* = 0.932, and *SRMR* = 0.040. The scale demonstrated good internal consistency in the present study (*Cronbach*’s *α* = 0.842).

#### Peer relationships

Peer relationships were assessed using the Peer Relationship Scale, originally developed by Asher et al. (1984) and revised for Chinese children by Zou (1998). The scale comprises 14 items and includes two subscales: peer acceptance and peer rejection. All items were rated on a 4-point Likert scale. Reverse-coded items were recoded prior to analysis, with higher scores indicating better peer relationship quality. A confirmatory factor analysis indicated an acceptable model fit, *χ*^2^(73) = 255.50, *p* < 0.001, *RMSEA* = 0.065 (90% CI [0.056, 0.073]), *CFI* = 0.921, *TLI* = 0.901, and *SRMR* = 0.043. The scale demonstrated good internal consistency in the present study (*Cronbach*’s *α* = 0.852).

#### Perceived teacher support

Perceived teacher support was assessed using the Student-Perceived Teacher Support Questionnaire (Babad, 1990), revised for Chinese children by Ouyang (2005). The scale consists of 19 items and captures three dimensions: academic support, emotional support, and competence support. All items were rated on a 4-point Likert scale. Based on preliminary confirmatory factor analyses, one item was excluded due to a low factor loading, resulting in an 18-item scale used in the final analyses. Composite scores were computed, with higher scores indicating greater perceived teacher support. A confirmatory factor analysis supported the three-factor structure, showing an acceptable model fit, *χ*^2^(128) = 361.24, *p* < 0.001, *RMSEA* = 0.055 (90% CI [0.048, 0.062]), *CFI* = 0.922, *TLI* = 0.907, and *SRMR* = 0.042. The scale demonstrated good internal consistency in the present study (*Cronbach*’s *α* = 0.865).

#### Mental health

Children’s mental health was measured using the 12-item General Health Questionnaire (GHQ-12; Goldberg et al., 1997), which assesses general psychological well-being and emotional distress. Items were rated on a 4-point Likert scale (1 = strongly disagree, 4 = strongly agree). After reverse-coded items were recoded, a total score was computed, with higher scores indicating better mental health. A confirmatory factor analysis supported the two-factor structure, showing an acceptable model fit, *χ*^2^(53) = 163.90, *p* < 0.001, *RMSEA* = 0.059 (90% CI [0.049, 0.069]), *CFI* = 0.942, *TLI* = 0.928, and *SRMR* = 0.039. The scale demonstrated good internal consistency in the present study (*Cronbach*’s *α* = 0.831).

### Statistical analysis

Data analyses were conducted in several steps. First, measurement invariance across migrant and non-migrant groups was examined to ensure the comparability of constructs.

Descriptive statistics, Pearson correlations, and multiple regression analyses were then conducted to examine the distributional properties of the variables and their interrelationships.

To further investigate the associations among parent–child communication, peer relationships, perceived teacher support, and children’s mental health, a latent variable structural equation model (SEM) was estimated to test the serial mediation model. In addition, a multi-group path analysis based on observed composite variables was performed to examine group differences and moderated mediation effects. Indirect effects were estimated using bootstrap procedures with 5,000 resamples. Statistical significance of indirect effects was evaluated using bias-corrected bootstrap 95% confidence intervals (Preacher and Hayes, 2008). Indirect effects were considered statistically significant when the confidence interval did not include zero.

Although students were nested within classes and schools, the potential impact of clustering was considered. However, due to the limited number of higher-level units and the primary focus on individual-level associations, the analyses were conducted at the individual level without applying multilevel modeling.

## Results

### Measurement invariance

To ensure comparability across groups, measurement invariance was first conducted to examine whether the measurement properties of parent–child communication, peer relationships, perceived teacher support, and mental health were equivalent across migrant and non-migrant children. Given the relatively large number of items for each construct, a parceling approach was adopted to improve model parsimony and estimation stability (Little et al., 2002).

Measurement invariance across migrant and non-migrant children was examined sequentially through configural, metric, and scalar models. The configural model demonstrated a good fit to the data (*CFI* = 0.961, *TLI* = 0.952, *RMSEA* = 0.035, 90% CI [0.029, 0.040], *SRMR* = 0.041), indicating that the overall factor structure was similar across groups. Constraining factor loadings to be equal across groups (metric invariance) resulted in minimal changes in model fit (Δ*CFI* < 0.01, Δ*RMSEA* = +0.001). Further constraining item intercepts (scalar invariance) also did not significantly worsen model fit (Δ*CFI* < 0.01, Δ*RMSEA* = +0.001). According to recommended criteria (Cheung and Rensvold, 2002), these results support scalar invariance across groups, suggesting that the constructs were measured in a comparable manner across migrant and non-migrant children, providing a valid basis for subsequent comparisons of structural associations.

### Group differences in descriptive statistics

Descriptive statistics for parent–child communication, peer relationships, perceived teacher support, and mental health are presented in Table 1. Skewness and kurtosis values for all variables fell within the range of ±1, indicating that the data approximated a normal distribution. Independent-samples *T*-tests were conducted to examine differences between migrant and non-migrant children across the four study variables. The results indicated that the two groups did not differ significantly in parent–child communication, peer relationships, perceived teacher support, or mental health (|*t*|s < 1.71, *p*s > 0.05) further confirming that the two groups were comparable on these variables at the mean level.

**Table 1:** Descriptive statistics and group differences.

Variable	*M* ± *SD*	Skew	Kurt	Migrant (*M ± SD*)	Non-migrant (*M ± SD*)	*t*	*p*
Parent–child communication	72.55 ± 11.13	−0.581	−0.003	72.79 ± 11.43	72.32 ± 10.86	0.514	0.607
Peer relationships	47.58 ± 6.83	−0.876	0.640	47.09 ± 7.33	48.05 ± 6.28	−1.713	0.087
Perceived teacher support	52.42 ± 9.95	−0.288	0.051	52.05 ± 10.75	52.78 ± 9.11	−0.892	0.373
Mental health	37.08 ± 7.03	−0.634	0.180	36.94 ± 7.54	37.21 ± 6.50	−0.461	0.645

*N* = 600 (migrant = 303, non-migrant = 297). All *p*-values are two-tailed.

### Group differences in multiple regression analyses

Prior to conducting multiple regression analyses, partial correlation analyses were performed separately for migrant and non-migrant children, controlling for gender and grade (see Table 2). The results indicated that, for both migrant and non-migrant children, parent–child communication, peer relationships, and perceived teacher support were all significantly and positively correlated with mental health (*r*s ≥ 0.299, *p*s < 0.001), suggesting that the variables were related but not excessively overlapping.

**Table 2:** Correlations among variables for migrant and non-migrant children.

Variable	1	2	3	4
1. Parent–child communication	–	0.391***	0.346***	0.552***
2. Peer relationships	0.473***	–	0.337***	0.380***
3. Perceived teacher support	0.412***	0.401***	–	0.299***
4. Mental health	0.591***	0.626***	0.406***	–

Correlations below the diagonal represent migrant children (*N* = 303), whereas correlations above the diagonal represent non-migrant children (The bold and italicized values (*N* = 297). *p < 0.05, **p < 0.01, ***p < 0.001.

Further comparisons of the correlation coefficients between the two groups revealed that the significant difference was for the association between peer relationships and mental health, which was significantly stronger in the migrant group (*r* = 0.626) than in the non-migrant group (*r* = 0.380; *Z* = 4.071, *p* < 0.001). For all other variable pairs, the correlations did not differ significantly between the two groups (*p*s > 0.05). These findings provide preliminary support for including these variables in subsequent regression and path analyses across groups.

Multiple regression analyses were then conducted separately for migrant and non-migrant children. Gender and grade were entered as control variables, while parent–child communication, peer relationships, and perceived teacher support were included as independent variables, with mental health as the dependent variable. Multicollinearity diagnostics indicated that variance inflation factor (VIF) values for all predictors were below 2, suggesting that multicollinearity was not a concern. The regression analysis results are shown in Table 3.

**Table 3:** Multiple regression analyses for migrant and non-migrant children.

Predictors	Model 1: Migrant children				Model 2: Non-migrant children			
	*b (SE)*	*β*	*t*	*p*	*b (SE)*	*β*	*t*	*p*
Grade	0.273 (0.408)	0.034	0.670	0.503	−0.005 (0.466)	−0.001	−0.010	0.992
Gender	−1.340 (0.661)	−0.103	−2.028	0.043	0.115 (0.758)	0.008	0.152	0.879
Parent–child communication	**0.211 (0.029)**	**0.352**	**7.369**	**<0.001**	**0.301 (0.035)**	**0.456**	**8.543**	**<0.001**
Peer relationships	**0.435 (0.049)**	**0.420**	**8.839**	**<0.001**	**0.179 (0.055)**	**0.174**	**3.270**	**0.001**
Perceived teacher support	**0.066 (0.033)**	**0.092**	**1.986**	**0.048**	0.058 (0.037)	0.083	1.598	0.111
	*F*(5, 297) = 62.942, *p* < 0.001, *R*^2^ = 0.514				*F*(5, 291) = 30.434, *p* < 0.001, *R*^2^ = 0.332			

Bold values indicate statistically significant coefficients (*p* < 0.05).

For migrant children, peer relationships showed the strongest association with mental health (*β* = 0.420, *t* = 8.839, *p* < 0.001), followed by parent–child communication (*β* = 0.352, *t* = 7.369, *p* < 0.001) and perceived teacher support (*β* = 0.092, *t* = 1.986, *p* = 0.048). The overall model was significant, *F*(5, 297) = 62.942, *p* < 0.001, and accounted for 51.4% of the variance in mental health.

In contrast, among non-migrant children, parent–child communication showed the strongest association with mental health (*β* = 0.456, *t* = 8.534, *p* < 0.001), followed by peer relationships (*β* = 0.174, *t* = 3.270, *p* = 0.001). Perceived teacher support was not significantly associated with mental health (*β* = 0.083, *t* = 1.598, *p* = 0.111). The overall model was significant, *F*(5, 291) = 30.434, *p* < 0.001, explaining 33.2% of the variance in mental health.

Tests of regression coefficients indicated that peer relationships were more strongly associated with mental health among migrant children (Δ*b* = 0.256, *Z* = 3.476, *p* < 0.001); Parent–child communication was stronger among non-migrant children (Δ*b* = 0.090, *Z =* 1.980, *p* = 0.048). No group differences were observed for perceived teacher support (Δ*b* = 0.017, *Z* = 0.343, *p* = 0.732) or control variables.

Overall, parent–child communication was more strongly associated with mental health among non-migrant children, whereas peer relationships were more strongly associated with mental health among migrant children, although parent–child communication remained significant in both groups.

### Serial mediation analysis

To further examine the associations among parent–child communication, peer relationships, perceived teacher support, and children’s mental health, a structural equation model (SEM) was estimated using Mplus 8.3. A mediation model was specified in which peer relationships and perceived teacher support functioned as mediators linking parent–child communication to mental health. Latent constructs were represented using parceled indicators constructed based on measurement invariance across groups to ensure comparability as well as model parsimony and estimation stability. Indirect effects were evaluated using a bootstrap procedure with 5,000 resamples.

The model yielded a good fit to the data, *χ*^2^(310) = 813.814, *p* < 0.001; *CFI* = 0.926, *TLI* = 0.916; *RMSEA* = 0.052 (90% CI [0.048, 0.056]), *SRMR* = 0.057. These fit indices indicate that the hypothesized model adequately represented the observed data. In this model (see diagram in the attachment), parent–child communication was positively associated with both peer relationships (*β* = 0.597, *p* < 0.001) and perceived teacher support (*β* = 0.355, *p* < 0.001). Peer relationships, in turn, were positively associated with perceived teacher support (*β* = 0.301, *p* < 0.001) and with mental health (*β* = 0.316, *p* = 0.001). However, the path from perceived teacher support to mental health was not significant (*β* = 0.101, *p* = 0.341).

Bootstrap analyses (see Table 4) further revealed that the indirect effect of parent–child communication on mental health through peer relationships alone was significant (Indirect 1: *b* = 0.261, *SE* = 0.087, 95% CI [0.095, 0.430], *β* = 0.189), accounting for 22.63% of the total effect. In contrast, the indirect effect through teacher support alone was not significant (Indirect 2: *b* = 0.050, *SE* = 0.063, 95% CI [−0.048, 0.208], *β* = 0.036), nor the serial indirect effect through peer relationships and then teacher support (Indirect 3: *b* = 0.025, *SE* = 0.030, 95% CI [−0.018, 0.101], *β* = 0.018), because the 95% CIs contained zero.

**Table 4:** Mediated effects of serial mediation.

Mediation pathways	*b*	*SE*	95%CI	*β*	VAF (%)
Indirect 1	**0.261**	**0.087**	**[0.095, 0.430]**	**0.189**	**22.63%**
Indirect 2	0.050	0.063	[−0.048, 0.208]	0.036	4.31%
Indirect 3	0.025	0.030	[−0.018, 0.101]	0.018	2.16%
Total indirect effect	**0.337**	**0.127**	**[0.094, 0.607]**	**0.243**	**29.10%**
Direct effect	**0.821**	**0.179**	**[0.527, 1.226]**	**0.592**	**70.90%**
Total effect	**1.157**	**0.179**	**[0.854, 1.551]**	**0.835**	**100.00%**

Indirect 1 = parent–child communication → Peer relationships → Mental health; Indirect 2 = parent–child communication → Perceived teacher support → Mental health; Indirect 3 = parent–child communication → Peer relationships → Perceived teacher support → Mental health. Bold values indicate statistically significant coefficients (*p* < 0.05).

The total indirect effect was significant (*b* = 0.337, *SE* = 0.127, 95% CI [0.094, 0.607], *β* = 0.243), explaining 29.10% of the total effect. The direct effect of parent–child communication on mental health remained significant (*b* = 0.821, *SE* = 0.179, 95% CI [0.527, 1.226], *β* = 0.592), and the total effect was also significant (*b* = 1.157, *SE* = 0.179, 95% CI [0.854, 1.551], *β* = 0.835), suggesting that indirect pathways, particularly peer relationships, played a substantial indirect role among Chinese children.

#### Multi-group path analysis

**Figure 1:**
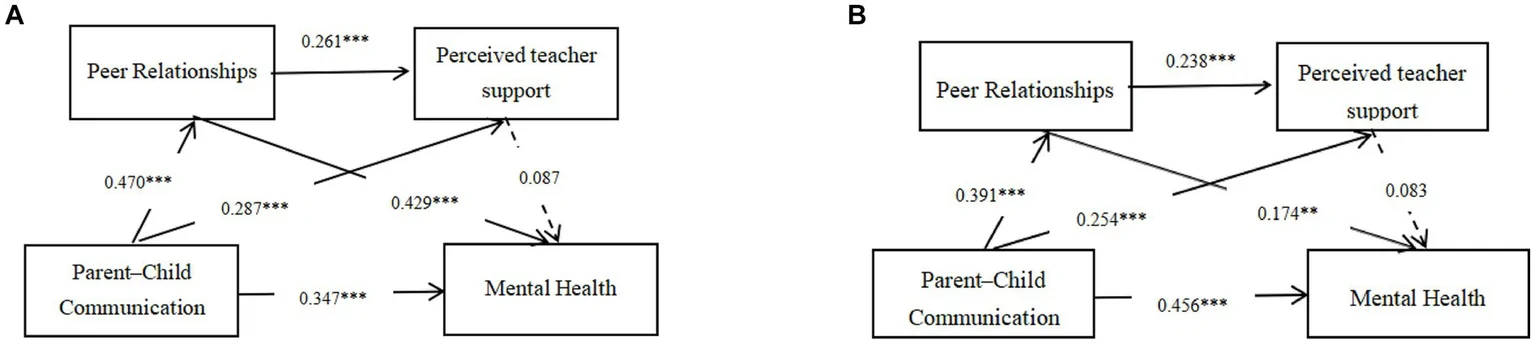
**(A)** Serial mediation model for migrant children. **(B)** Serial mediation model for non-migrant children. **p < 0.01, ***p < 0.001.

Given that measurement invariance was supported across groups, multi-group path analysis was conducted to examine whether migration experience moderated the structural effects and mediation pathways between migrant and non-migrant children. Considering model parsimony and the need for direct comparability of structural coefficients across groups, a multi-group path analysis based on observed variables was employed. Bootstrap procedures with 5,000 resamples were used to estimate group-specific indirect effects. Indirect effects are presented in Table 5 and visualized in Figures 1A,B.

**Table 5:** Comparison of structural paths between migrant and non-migrant children.

Path	Migrant children				Non-migrant children				Δ*b*	95%CI
	*b* (*SE*)	95%CI	*β*	*p*	*b* (*SE*)	95%CI	*β*	*p*		
Path A1	0.272 (0.034)	[0.206, 0.339]	0.470	<0.001	0.251 (0.039)	[0.177, 0.328]	0.391	<0.001	0.021	[−0.080, 0.122]
Path A2	0.378 (0.092)	[0.203, 0.558]	0.261	<0.001	0.349 (0.089)	[0.174, 0.521]	0.238	<0.001	0.029	[−0.222, 0.280]
Path A3	0.241 (0.059)	[0.124, 0.352]	0.287	<0.001	0.239 (0.057)	[0.130, 0.353]	0.254	<0.001	0.002	[−0.159, 0.163]
Path A4	0.062 (0.032)	[−0.001, 0.126]	0.087	0.053	0.058 (0.038)	[−0.016, 0.131]	0.083	0.121	0.004	[−0.093, 0.101]
Path A5	0.444 (0.054)	[0.342, 0.556]	0.429	<0.001	0.179 (0.059)	[0.066, 0.296]	0.174	0.002	**0.265**	**[0.120, 0.410]**
Direct effect	0.208 (0.033)	[0.144, 0.276]	0.347	<0.001	0.301 (0.037)	[0.230, 0.376]	0.456	<0.001	−0.093	[−0.190, 0.004]
Indirect 1	0.121 (0.020)	[0.086, 0.166]	0.202	–	0.045 (0.017)	[0.016, 0.084]	0.068	–	**0.076**	**[0.025, 0.132]**
Indirect 2	0.015 (0.009)	[0.001, 0.036]	0.025	–	0.014 (0.009)	[−0.002, 0.035]	0.021	–	0.001	[−0.023, 0.028]
Indirect 3	0.006 (0.004)	[0.001, 0.016]	0.011	–	0.005 (0.004)	[−0.001, 0.015]	0.008	–	0.001	[−0.009, 0.012]
Total indirect effect	0.142 (0.022)	[0.105, 0.189]	0.238	–	0.064 (0.021)	[0.026, 0.109]	0.097	–	**0.078**	**[0.018, 0.138]**
Total effect	0.350 (0.035)	[0.281, 0.418]	0.585	–	0.365 (0.035)	[0.294, 0.433]	0.553	–	−0.015	[−0.112, 0.082]

Path A1 = parent–child communication → Peer relationships; Path A2 = Peer relationships → Teacher support; Path A3 = parent–child communication → Teacher support; Path A4 = Teacher support → Mental health; Path A5 = Peer relationships → Mental health; Indirect 1 = parent–child communication → Peer relationships → Mental health; Indirect 2 = parent–child communication → Perceived teacher support → Mental health; Indirect 3 = parent–child communication → Peer relationships → Perceived teacher support → Mental health. Bold values indicate statistically significant coefficients (*p* < 0.05).

#### Serial mediation model for migrant children

Among migrant children (Figure 1A), parent–child communication was significantly associated with peer relationships (*β* = 0.470, *p* < 0.001), and peer relationships were positively associated with perceived teacher support (*β* = 0.261, *p* < 0.001). Parent–child communication also had a significant direct association with perceived teacher support (*β* = 0.287, *p* < 0.001). Regarding mental health, peer relationships showed a significant positive association (*β* = 0.429, *p* < 0.001), whereas perceived teacher support was not significance (*β* = 0.087, *p* = 0.053). Parent–child communication remained significantly associated with mental health (*β* = 0.347, *p* < 0.001).

Bootstrap analyses indicated that the indirect effect through peer relationships alone was significant (*b* = 0.121, *SE* = 0.020, 95% CI [0.086, 0.166], *β* = 0.202). The indirect effect through perceived teacher support alone was significant (*b* = 0.015, *SE* = 0.009, 95% CI [0.001, 0.036], *β* = 0.025), and the serial mediation pathway was also significant (*b* = 0.006, *SE* = 0.004, 95% CI [0.001, 0.016], *β* = 0.011), as the 95% bootstrap confidence intervals did not include zero. However, the effects through perceived teacher support was small and are therefore interpreted cautiously.

The total indirect effect was significant (*b* = 0.142, *SE* = 0.022, 95% CI [0.105, 0.189], *β* = 0.238), and the direct effect remained significant (*b* = 0.208, *SE* = 0.033, 95% CI [0.144, 0.276], *β* = 0.347), indicating a partial mediation model. The total indirect effect accounted for approximately 40.7% of the total effect (0.238/0.585), suggesting that indirect pathways—particularly those involving peer relationships—played a substantial role among migrant children.

### Serial mediation model of non-migrant children

Among non-migrant children (Figure 1B), parent–child communication was significantly associated with peer relationships (*β* = 0.391, *p* < 0.001), which in turn significantly predicted perceived teacher support (*β* = 0.238, *p* < 0.001). Parent–child communication also had a significant direct association with perceived teacher support (*β* = 0.254, *p* < 0.001).

With regard to mental health, peer relationships had a significant positive effect (*β* = 0.174, *p* = 0.002), whereas the effect of perceived teacher support was not statistically significant (*β* = 0.083, *p* = 0.121). Parent–child communication remained significantly associated with mental health (*β* = 0.456, *p* < 0.001).

Bootstrap analyses showed that the indirect effect of parent–child communication on mental health through peer relationships alone was significant (*b* = 0.045, *SE* = 0.017, 95% CI [0.016, 0.084], *β* = 0.068). The indirect effect through perceived teacher support alone was not significant (*b* = 0.014, *SE* = 0.009, 95% CI [−0.002, 0.035], *β* = 0.021), nor the serial mediation pathway through peer relationships and perceived teacher support (*b* = 0.005, *SE* = 0.004, 95% CI [−0.001, 0.015], *β* = 0.008).

Overall, the total indirect effect was significant (*b* = 0.064, SE = 0.021, 95% CI [0.026, 0.109], *β* = 0.097), and the direct effect remained significant (*b* = 0.301, SE = 0.037, 95% CI [0.230, 0.376], *β* = 0.456), indicating a partial mediation model. The total indirect effect accounted for 17.5% of the total effect (0.097/0.553), suggesting that the association between parent–child communication and mental health among non-migrant children was primarily driven by the direct pathway.

### Comparisons of pathway between groups

Multi-group comparisons indicated that although the overall pattern of the model path was broadly similar across groups, several path coefficients differed significantly between migrant and non-migrant children (Table 5).

The effect of peer relationships on mental health (Path A5) was significantly stronger among migrant children than non-migrant children (Δ*b* = 0.265, 95% CI [0.120, 0.410]). The indirect effect linking parent–child communication to mental health through peer relationships alone (Indirect 1: Δ*b* = 0.076, 95% CI [0.025, 0.132]) and the total indirect effect (Δ*b* = 0.078, 95% CI [0.018, 0.138]) were significantly stronger among migrant children. No significant group differences were observed for the other paths or indirect effects (*ps* > 0.05).

## Discussion

Grounded in ecological systems theory (Bronfenbrenner, 1979), the present study examined the associations among parent–child communication, peer relationships, perceived teacher support, and children’s mental health, with a particular focus on whether these associative patterns differed between migrant and non-migrant children. Three findings stand out. First, parent–child communication was consistently associated with children’s mental health in both groups, underscoring the continuing importance of the family microsystem. Second, peer relationships played a more prominent role among migrant children: peer relationships were more strongly associated with mental health in this group, and the indirect pathway linking parent–child communication to mental health through peer relationships was also stronger among migrant children. Third, perceived teacher support showed weak and generally non-significant associations with mental health in both groups. Taken together, these findings suggest that migration may not necessarily be associated with lower average levels of support or poorer mental health, but it may be associated with differences in how family- and school-based support systems are organized and linked to children’s adjustment.

### No significant mean difference between migrant and non-migrant children

A notable finding of the present study is that migrant and non-migrant children did not differ significantly in mean levels of parent–child communication, peer relationships, perceived teacher support, or mental health. This finding differs from earlier studies that have suggested migrant children as consistently disadvantaged in family support, peer integration, and psychological adjustment (Li et al., 2016; Li and Jiang, 2018; Liu et al., 2022; Tang et al., 2024; Xiong and Ye, 2011). One plausible interpretation is that these null differences reflect a degree of convergence in children’s support experiences and mental health outcomes. From a chronosystem perspective, broader sociohistorical changes—such as the expansion of urban educational access and inclusion policies for migrant children—may have reduced some of the disparities reported in earlier work. In this sense, migration-related disadvantage may be less visible at the level of mean scores than in the past.

At the same time, this interpretation should be made cautiously. The present study did not directly assess policy exposure, school inclusion practices, migration duration, or other contextual indicators that would allow a stronger test of the “convergence” explanation. It is therefore equally important to consider measurement and sampling explanations. The scales used in this study captured broad perceptions of family communication, peer relationships, teacher support, and general mental health, but may have been less sensitive to more subtle migration-related stressors, exclusion experiences, or differences in support quality. In addition, the sample was drawn from three public primary schools in one northern Chinese city, which may represent relatively integrated school settings rather than the full range of migrant children’s experiences. Accordingly, the present finding should not be interpreted as definitive evidence that migrant and non-migrant children are now equivalent in support or mental health. Rather, it suggests that mean-level group comparisons may no longer be sufficient on their own, and that greater insight may come from examining whether support systems function differently across migration contexts.

### Parent–child communication as a central correlate of mental health

A central finding of the present study is that parent–child communication was consistently associated with children’s mental health in both migrant and non-migrant groups. This result underscores the enduring importance of the family microsystem in children’s psychological adjustment and is broadly consistent with prior research showing that open, supportive, and emotionally responsive communication within the family is associated with better emotional regulation, stronger security, and fewer internalizing difficulties during childhood and adolescence (Barnes and Olson, 1985; Li and Mo, 2024; Wagner et al., 2025; Yuan et al., 2024). Within the ecological system framework, the family remains the most proximal and developmentally enduring context (Bronfenbrenner, 1979), and the present findings suggest that this foundational role is preserved across migration contexts.

This result further suggests that parent–child communication may operate not only as a direct correlate of mental health, but also as an important relational foundation from which children engage with other social contexts. In ecological terms, family communication may shape how children interpret, initiate, and maintain social interactions beyond the home, thereby linking the family microsystem to children’s functioning in peer and school settings (Delgado et al., 2022; Sroufe, 2005). This interpretation is consistent with the indirect pathways observed in the present study, particularly the pathway through peer relationships, and highlights the possibility that family processes remain central even when their effects are partly transmitted through other microsystems.

Although the direct association between parent–child communication and mental health appeared marginally stronger among non-migrant children, this difference was not statistically robust. At most, the pattern may tentatively suggest that in relatively stable family environments, the influence of parent–child communication on children’s mental health may remain more directly expressed, whereas in migrant children, some of this influence may be channeled through adaptation-related peer processes. However, because the confidence interval for the group difference included zero, the more defensible conclusion is that parent–child communication represents a common and important correlate of mental health across both groups, while any difference in its relative strength remains uncertain and requires replication.

### Peer relationships as a particularly salient pathway for migrant children

Against shared family foundation, the clearest migration-related difference in the present study concerned peer relationships. Compared with non-migrant children, migrant children showed a stronger association between peer relationships and mental health, as well as a stronger indirect pathway from parent–child communication to mental health through peer relationships. These findings suggests that while parent–child communication remains important for all children, peer relationships may assume heightened significance for migrant children, both as a direct correlate of psychological adjustment and as a pathway through which family experiences are linked to mental health.

One plausible interpretation is that peer relationships function as compensatory social resources in the context of migration (Butler et al., 2022). Migration may disrupt established social routines, reduce continuity in neighborhood and school-based relationships, and place pressure on family time and emotional availability (Liu et al., 2022; Tang et al., 2024). Under these conditions, peer networks may become a particularly accessible source of belonging, validation, and day-to-day emotional support (Allen et al., 2022; Meehan et al., 2003). From this perspective, positive peer relationships may help buffer the psychosocial demands of adaptation, especially when children are navigating unfamiliar school and community environments. The finding that parent–child communication was associated with mental health through peer relationships is consistent with this interpretation, insofar as supportive family communication may facilitate the development of trust, social competence, and relational security that can be carried into peer interactions.

A complementary explanation is that migration heightens the ecological salience of the peer microsystem. For migrant children, adjustment to a new urban setting often unfolds in peer contexts, where issues of belonging, acceptance, identity, and social integration are negotiated on a daily basis. In this sense, peer experiences may carry greater emotional weight not only because peers provide support, but also because peer acceptance or exclusion may become especially consequential for adaptation. This interpretation is broadly consistent with previous research suggesting that migrant children may be more vulnerable to peer difficulties, including social exclusion, friendship instability, and challenges in school belonging (Li and Jiang, 2018; Zhu et al., 2013). Accordingly, the stronger peer pathway observed in the present study may reflect both greater reliance on peers as a resource for adaptation and greater sensitivity to peer-related stressors. Although the present data cannot distinguish between these possibilities, they converge on the same substantive implication: among migrant children, peer relationships appear to be a particularly important pathway through which social experiences become linked to mental health.

From an ecological systems perspective, this finding also highlights the importance of mesosystem processes (Bronfenbrenner, 1979; Cox and Paley, 2003). The stronger indirect pathway from parent–child communication to mental health through peer relationships suggests that, for migrant children in particular, functioning within the family microsystem may be more tightly linked to functioning within the peer microsystem. Migration may therefore matter not only because it changes children’s social circumstances, but because it may reorganize the interconnections among support systems that shape psychological adjustment (Tudge et al., 2022; Lou et al., 2025).

### Perceived teacher support shows limited associations

Although the bootstrap confidence intervals provided limited evidence for indirect pathways involving perceived teacher support, these effects were small. Moreover, comparable indirect effects were not observed in the full sample or among non-migrant children. These findings suggest that the role of perceived teacher support was limited and not sufficiently robust to constitute a primary pathway linking parent–child communication with children’s mental health.

This suggests that not all elements of the school microsystem are equally linked to children’s mental health, even when they occupy the same general context. One possible explanation is that the role of teacher support in upper primary school may be more indirect and context-dependent than the present measure was able to capture. The scale assessed children’s overall perceptions of teacher support, including academic support, emotional support, and competence support (Ouyang, 2005). Still, it may not have captured the specific dimensions of school support most relevant to psychological adjustment in upper primary school. It is possible that broader school-climate indicators, such as classroom belonging and emotional safety (Chen et al., 2025), would show stronger associations with mental health than general perceived teacher support alone.

A second explanation may relate to the increasing achievement orientation of urban basic education in the context of rapid economic change. As urban schooling has become more strongly shaped by academic competition and pressure for educational attainment, teachers may be perceived primarily as academic instructors and authority figures, with academic performance prioritized over emotional well-being (Wu et al., 2025). Under such conditions, emotional support may be sought more readily from peers or family members than from teachers. If children interpret “teacher support” largely in terms of academic guidance rather than emotional availability, its association with mental health may naturally be weaker. Future work would benefit from distinguishing between instructional support, emotional support, and broader classroom relational climate when examining the role of teachers in migrant and non-migrant children’s adjustment.

### An integrated ecological systems interpretation

Taken together, the findings suggest a differentiated ecological picture of children’s mental health. The family microsystem, indexed here by parent–child communication, remained an important correlation of mental health for both migrant and non-migrant children. The peer microsystem, however, appears to take on heightened significance in the migration context, serving not only as a direct correlate of mental health but also as a pathway linking family communication to mental health. By contrast, teacher support dose not appear to function as an equally salient pathway for mental health in this sample.

This pattern extends ecological systems theory in two ways. First, it supports the idea that migration can be understood not merely as a demographic characteristic, but as a chronosystem-related life transition that may reorganize children’s developmental ecology. Importantly, the present findings suggest that such reorganization may be reflected less in mean-level difference than in the relative salience and interdependence of support pathways. Second, the results illustrate that microsystems should not be treated as internally homogeneous. Within the school microsystem, peer relationships and teacher support appear to have different psychological functions, and these functions may vary in their relevance to mental health across developmental stages and migration contexts.

### Implications for practices

The present findings have several implications for intervention and school practice. First, because parent–child communication was consistently associated with mental health in both groups, family-based efforts to strengthen communication remain important for all children. Programs that support parents in emotional communication, active listening, and conflict resolution may be beneficial regardless of migration status (Chen et al., 2026).

Second, the heightened salience of peer relationships among migrant children suggests that school-based mental health support should not focus solely on individual coping or teacher-student relationships. Interventions that foster inclusive peer environments—such as cooperative learning, peer mentoring, social-skills training, and anti-exclusion practices—may be particularly relevant for migrant children’s psychological adjustment (Kim et al., 2026; Wentzel et al., 2016). These approaches may be especially valuable in contexts where family support is strained by migration-related demands or where children are navigating the social challenges of adaptation to a new school environment.

At the same time, the weak direct role of perceived teacher support should not be taken to mean that teachers are unimportant. Rather, it may indicate that teachers contribute to mental health most effectively by shaping the peer ecology of the classroom: promoting belonging, reducing exclusion, and creating a supportive relational climate. In this sense, teacher practices may matter less as a direct emotional resource and more as a contextual force that influences how peer support unfolds (Gest and Rodkin, 2011; Zhang and Liu, 2025).

### Limitations and future directions

This study has several limitations. First, the cross-sectional design precludes causal or temporal inference. Although the mediation model estimated statistical direct and indirect effects in a statistical sense, these pathways should be interpreted as associative rather than causal. Longitudinal and cross-lagged designs will be needed to determine whether parent–child communication prospectively predicts peer relationships and mental health, or whether these associations are bidirectional.

Second, migration status was operationalized as a binary group variable, and several potentially relevant background factors, such as socioeconomic status, parental education, family structure, and length of residence, were not available in the present dataset. As a result, the present study could not capture heterogeneity within the migrant group or fully account for contextual factors that may shape children’s support experiences and mental health. Future research should incorporate more fine-grained migration-related and family contextual indicators to better clarify the conditions under which different support pathways become salient.

Third, all variables were assessed through child self-report, and the sample was drawn from three public primary schools in one northern Chinese city. These features raise concerns about shared method variance and limit the generalizability of the findings. Future studies would benefit from multi-informant designs and more diverse samples across regions and school contexts.

## Conclusion

The findings indicate that parent–child communication emerged as a consistent correlate of mental health in both migrant and non-migrant children, whereas peer relationships emerged as a particularly salient correlate of mental health only among migrant children, both directly and indirectly. By contrast, perceived teacher support showed limited direct associations with mental health in this sample. Overall, these findings extend ecological systems theory, underscore the importance of examining the configuration of family and school support systems across migration contexts, and highlight the importance of strengthening parent–child communication while also supporting peer relationships, particularly among migrant children.

## Data Availability

The original contributions presented in the study are included in the article/supplementary material, further inquiries can be directed to the corresponding author.
